# Inflammation mediates approximately one quarter of excess relative all-cause mortality in persons with rheumatoid arthritis: the Trøndelag Health Study

**DOI:** 10.1038/s41598-022-21977-9

**Published:** 2022-11-03

**Authors:** Vibeke Videm, Ingrid Sæther Houge, Marthe Halsan Liff, Mari Hoff

**Affiliations:** 1 Department of Clinical and Molecular Medicine, NTNU-Norwegian University of Science and Technology, Trondheim, Norway; 2 Department of Immunology and Transfusion Medicine, St. Olavs University Hospital, Trondheim, Norway; 3 Department of Clinical and Molecular Medicine, Lab Center 3 East, St. Olavs University Hospital, 7006 Trondheim, Norway; 4 Department of Rheumatology, Levanger Hospital, Nord-Trøndelag Hospital Trust, Levanger, Norway; 5 Clinic of Orthopedics, Rheumatology and Dermatology, St. Olavs University Hospital, Trondheim, Norway; 6 Department of Neuromedicine and Movement Science, NTNU-Norwegian University of Science and Technology, Trondheim, Norway; 7 Department of Rheumatology, St. Olavs University Hospital, Trondheim, Norway

**Keywords:** Rheumatology, Risk factors

## Abstract

Inflammation may contribute to excess mortality in rheumatoid arthritis (RA) patients. We investigated associations to all-cause mortality of the inflammation markers high-sensitivity C-reactive protein (CRP), lactoferrin (neutrophil activation marker), and neopterin (monocyte activation marker). From the population-based Trøndelag Health Study (3rd wave 2006–2008), 316 RA patients and 43,579 controls were included. Lactoferrin and neopterin were quantified in a nested cohort (n = 283 RA patients, n = 3698 controls). Follow-up was until death found by linkage to the Norwegian Cause of Death Registry or 31.12.2018. All-cause mortality was analyzed using Cox regression and Cox regression-based mediation analysis. Having RA (hazard ratio (HR): 1.25, 95%CI: 1.00, 1.56, p = 0.048), and CRP ≥ 3 mg/L (HR: 1.50, 95%CI: 1.41, 1.60, p < 0.001) were associated with all-cause mortality. The overall excess relative mortality risk of having RA was 38%. CRP ≥ 3 mg/L mediated approximately 1/4 of this risk (p < 0.001). In the nested cohort, CRP ≥ 3 mg/L (HR: 1.51, 95%CI: 1.26, 1.80, p < 0.001) and neopterin (HR: 1.17, 95%CI: 1.01, 1.36, p = 0.031) were associated with all-cause mortality. In conclusion, CRP levels ≥ 3 mg/L mediated approximately a quarter of the 38% excess relative all-cause mortality risk associated with RA. Using definitions of RA remission with emphasis both on joint status and the level of general inflammation may help guide the most efficient treatment regimens.

## Introduction

Rheumatoid arthritis (RA) is a common chronic inflammatory joint disease with persistent synovitis and systemic inflammation^1^. Other systemic effects and extraarticular manifestations are common, such as fatigue, depression, osteoporosis, cardiovascular disease (CVD), pulmonary disease, renal disease, and malignancies^1^.

Several recent studies have confirmed increased mortality rates in persons with RA compared to control populations or the general population^2–7^, even if not all have confirmed this finding^8^. The most important causes of death in persons with RA are circulatory diseases including ischemic heart disease and cerebrovascular diseases, cancer, and respiratory diseases including chronic obstructive pulmonary disease and interstitial lung disease^2,4,9^.

Various underlying reasons have been stated for the increased mortality rates in persons with RA, including an unfavorable cardiovascular (CV) risk factor profile, inflammation, life-style factors such as smoking and low cardiorespiratory fitness, and effects of medication^6,10–12^. Inflammation may be a central common element leading to premature death, partly because of accelerated atherosclerosis^11,13^. Several classical risk factors are associated with inflammation, including hypertension, hyperglycemia, and hypercholesterolemia^13^.

Biomarkers of inflammation have been suggested for risk stratification related to CVD and their association with future mortality risk is well established^14^. In the present study, we rather sought to use biomarkers to quantify associations of inflammation with all-cause mortality in RA patients compared to controls. Such quantification is possible using mediation analysis to investigate excess relative mortality in RA.

It is impossible to find one biomarker that covers all aspects of inflammation, which is a very complex process involving many mediators and cells, including monocytes/macrophages, neutrophils, and T-cells. For the present study, we therefore investigated three biomarkers related to different aspects of inflammation: C-reactive protein measured in a high-sensitivity assay (CRP), lactoferrin, and neopterin. They were selected for the following reasons: CRP is a common general marker of inflammation. It is released from the liver following stimulation by interleukin-6, and is produced by adipose tissue. CRP is associated with many endpoints including death and is used to quantify disease activity in RA^1,14^. Lactoferrin in serum is released from activated neutrophils and has diverse immunomodulatory and anti-inflammatory properties^15^. Concentrations are elevated in persons with significant coronary artery disease^16^ and lactoferrin predicts fatal ischemic heart disease in individuals with diabetes^17^. Neopterin is produced by macrophages and activated endothelial cells when stimulated by interferon-gamma from T-cells^18^. Neopterin is found in high concentrations in atherosclerotic plaques, where it probably acts as a compensatory inhibitor of proatherogenic effects^18,19^. Neopterin is also a marker of atherosclerotic plaque instability^20^.

The hypothesis for the present study was that biomarkers of inflammation representing different aspects of this complex process could act as proxies to quantify the importance of inflammation related to excess mortality in persons with RA compared to controls. The aims were therefore to investigate associations of CRP, lactoferrin, and neopterin to all-cause mortality in RA patients and controls in a large population-based study, including the use of mediation analysis.

## Methods

The Trøndelag Health Study (HUNT) is a population-based open cohort study performed in the northern part of Trøndelag county in Norway. All inhabitants aged ≥ 20 years are invited. The present study included participants from the 3rd wave, HUNT3 (2006–2008, participation rate ~ 54%). Data included measurements, results from a non-fasting blood sample, and answers to questionnaires. Serum samples stored at − 70 °C were provided from the HUNT Research Center. Written informed consent was obtained upon HUNT participation. The present study complies with the Declaration of Helsinki and was approved by the Norwegian Data Inspectorate and the Regional Committee for Medical and Health Research Ethics (#26264), as part of the HUNT project HuLARS (**HU**NT **L**ongitudinal **A**nklylosing spondylitis and **R**heumatoid arthritis **S**tudy).

**Figure 1:**
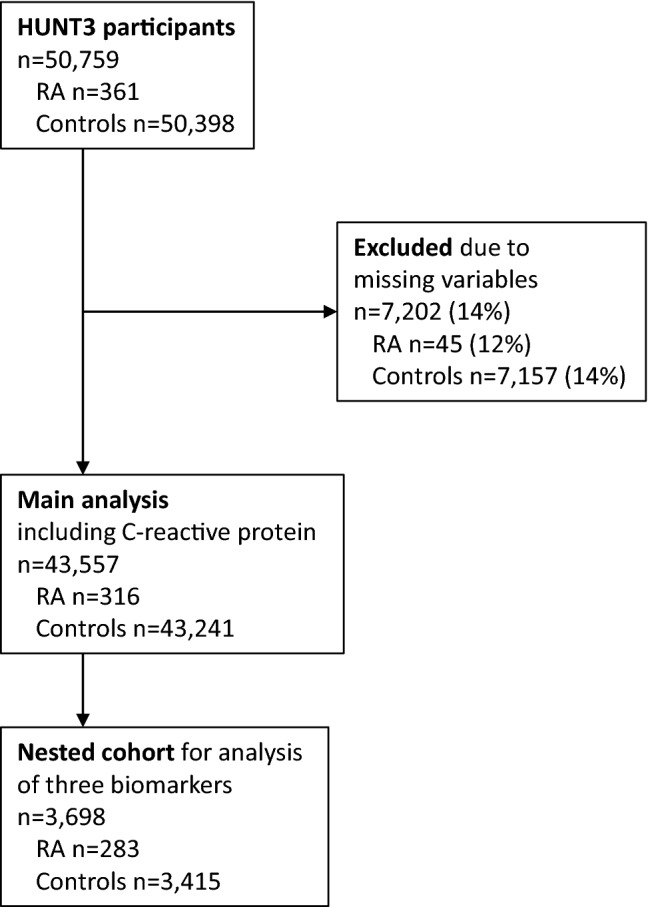
Participant inclusion and exclusion. Participants in the main analysis including C-reactive protein, and the nested cohort study including the biomarkers C-reactive protein, lactoferrin, and neopterin. Missing variables: C-reactive protein (1.5%), body mass index (0.8%), total cholesterol (2.9%), HDL cholesterol (2.9%), smoking (11.3%). HUNT3; 3rd wave of the Trøndelag Health Study (2006–2008).

RA diagnoses were ascertained for HuLARS by case file review^21^, based on the 2010 American College of Rheumatology/European League Against Rheumatism (EULAR) classification criteria^22^. All participants not fulfilling the RA diagnostic criteria were defined as controls, without exclusion of participants with other forms of arthritis due to missing information. It was also noted whether the RA patients were seropositive (i.e., positive for IgM rheumatoid factor and/or anti-citrullinated protein peptide antibodies). Figure 1 shows inclusion and exclusion to the present study, which had 2 parts: (1) Main study comparing all-cause mortality in RA patients and controls using CRP as biomarker of inflammation, and (2) Nested cohort study also including lactoferrin and neopterin as further biomarkers of inflammation.

The nested cohort was originally selected for a planned study where HUNT3 participants with self-reported RA or ankylosing spondylitis were 1:1 age- and gender-matched with a randomly selected participant without these self-reported diagnoses. That study was never completed, but serum from 3698 HUNT3 participants were available for the present study, comprising n = 283 persons with validated RA and n = 3415 persons without validated RA or ankylosing spondylitis. The cohort was no longer matched because many persons with self-reported arthritis had false-positive diagnoses^21^. Due to the low level of missingness in the main study, cases with complete data for the necessary variables were analyzed (Fig. 1).

CRP was analyzed by the HUNT Research Center shortly after HUNT3 participation using the Vario high-sensitivity kit (measurement range: 0.1–160.1 mg/L) on an Architect ci8200 instrument (Abbott Clinical Chemistry, Lake Forest, IL, USA). Lactoferrin was analyzed in a previously described in-house enzyme immunoassay^23^ using rabbit anti-human lactoferrin antibodies (Agilent, Santa Clara, CA, USA). Neopterin was analyzed using a commercial kit (Genway, San Diego, CA, USA).

All-cause mortality and causes of death were found through linkage of the study participants with the Norwegian Cause of Death Registry, which has > 99% coverage for Norwegian citizens, including those living abroad. Follow-up was from the inclusion date in HUNT3 until death or 31 December 2018, whichever came first.

### Statistics

Data were analyzed using Stata (version 16.1, StataCorp, College Station, TX, USA). Two-tailed P-values < 0.05 were considered statistically significant. Descriptive statistics are given as number (%) or median (95% CI) due to non-normal distributions in histograms of most variables. RA patients and controls were compared using the Chi-square test or Mann–Whitney U-test. Correlations among the three biomarkers at baseline were assessed using Spearman's rho. The binomial distribution was used to calculate 95% confidence intervals for mortality rates.

Mortality was first analyzed in several steps using Cox regression. Observation time started on the date of inclusion in HUNT3. Age, a very strong predictor of death was the time variable, ensuring that participants were always compared to controls of the same age. Furthermore, this design avoids problems with non-proportional hazards which often ensue with long observation times if age is used as an adjustment covariate instead of as the time variable. All models were stratified on sex, allowing for different baseline hazards in women and men.

In the main study, the Step1 models included RA (no/yes, Step1a) or CRP (< 3 mg/L vs. ≥ 3 mg/L, Step1b). CRP concentrations were highly skewed, and model fit using logarithmic transformation was not acceptable. Dichotomization was therefore preferred for two reasons: model fit was good, and the final model could be directly tested in mediation analysis (see below). The cut-off at 3 mg/L was chosen because this has previously been used to define "high risk" of CVD^14^. The Step2 models included either RA (Step2a) or CRP (Step2b) with adjustments for body mass index, diabetes (no/yes; self-reported diabetes and/or use of antidiabetic medication and/or non-fasting glucose concentration at HUNT3 > 11 mmol/L), hypertension (no/yes; systolic blood pressure ≥ 140 mmHg and/or diastolic blood pressure ≥ 90 mmHg and/or self-reported use of antihypertensive medication), smoking (self-reported never, previous, or present smoker), and total cholesterol. The Step3 model included both RA and CRP with the same adjustments. To assess potential bias due to dichotomization of CRP concentrations at 3 mg/L, a Step3 sensitivity analysis was performed using 5 mg/L as cut-off instead. Another sensitivity analysis included RA and CRP in the same model, but without the adjustments mentioned above. A second Step3 sensitivity analysis included further adjustments for previous self-reported cancer or chronic respiratory disease (asthma/chronic obstructive pulmonary disease).

In the nested cohort study, the Step4 models included either RA (Step4a), CRP (Step4b), lactoferrin concentrations (Step4c), or neopterin concentrations (Step4d). Both lactoferrin and neopterin concentrations were highly skewed and were transformed using natural logarithms to achieve proportional hazards. Dichotomization was not necessary to this end. The Step5 model included RA and all 3 biomarkers of inflammation and was adjusted as described above. Hazard ratios (HR) of the models are given with robust 95% confidence intervals (CI). Model fit including proportionality of hazards and the appropriate functional form of continuous variables was assessed using Schoenfeld and Martingale residuals, respectively. Models within the main study or within the nested cohort study were compared using the Akaike (AIC) and Bayesian information criteria (BIC), where a smaller numerical value indicates better fit.

**Figure 2:**
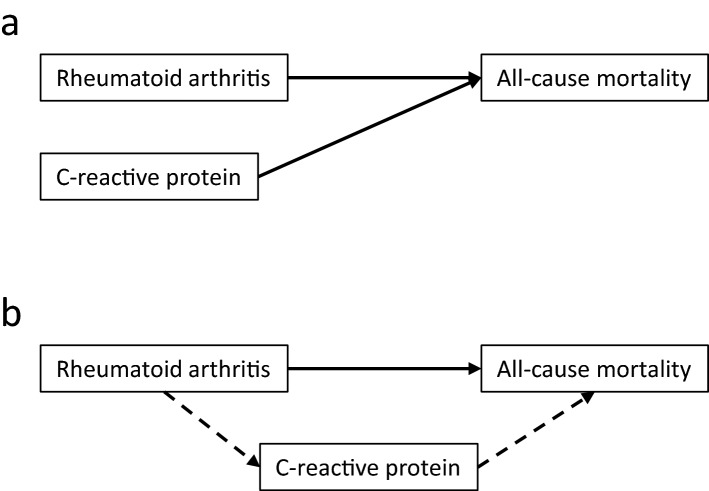
Analytical models. Panel a: In Cox regression, the independent associations with all-cause mortality of each of the explanatory variables presence of rheumatoid arthritis and C-reactive protein concentration ≥ 3 mg/L were investigated. Panel b: In the mediation analysis, the direct association with all-cause mortality of rheumatoid arthritis was investigated (), as well as the indirect effect of rheumatoid arthritis mediated by inflammation, using C-reactive protein concentration ≥ 3 mg/L as biomarker (). Both final models were adjusted for body mass index, diabetes, hypertension, smoking, and total cholesterol.

To investigate whether the effect of RA on mortality was mediated by inflammation, Cox regression-based mediation analysis was then performed in the main study cohort, using the Stata package med4way^24^. Figure 2 illustrates differences in modeling strategies using multivariable Cox regression and mediation analysis. Cox regression quantifies the independent parts of the association between RA or CRP on mortality. The change in HR for RA or CRP from Step1a or Step1b to Step2a or Step2b, respectively, shows how much each association overlaps with those of the added adjustment variables. A smaller HR may mean that part of the effect was mediated by the adjustment variables, which is relevant because some of the classical risk factors are associated with inflammation. However, Cox regression does not formally test this hypothesis. Similarly, the change in HR for RA or CRP from Step2a or Step2b to the common fully adjusted Step3 model, shows how much their associations overlap and thus whether part of the effect of RA may be mediated by CRP. Mediation analysis, on the other hand, formally tests the hypothesis that the effect of RA on mortality was mediated by CRP (Fig. 2b). Mediation analysis was not performed in the nested cohort study due to study size and inclusion of 3 inflammatory biomarkers. The number of RA patients was too low to perform a separate analysis in seropositive patients only.

## Results

Table 1 shows participant characteristics. In the main study, RA patients were older than controls and more often were female, had diabetes, hypertension, or were present/previous smokers. Patients also had higher median body mass index and CRP concentrations. In the nested cohort study, older age of RA patients and a higher frequency of hypertension and present/previous smoking were still notable. There were no significant differences in causes of death between RA patients and controls (Table 1). Concentrations of all three inflammation biomarkers were higher in the RA patients. Biomarker correlations were low or non-significant, indicating that they mainly represented different aspects of inflammation: CRP vs. lactoferrin; rho = 0.161, p < 0.001, CRP vs. neopterin; rho = 0.096, p < 0.001, lactoferrin vs. neopterin; rho = 0.005, p = 0.99. Table 1 gives observation times and mortality rates. Mortality rates were significantly higher in RA patients than controls (27% vs. 11%, p < 0.001).

**Table 1:** Participant characteristics.

Variable	Main study (n = 43,557)			Nested cohort study (n = 3698)		
	RA patients (n = 316)	Controls (n = 43,241)	P-value	RA patients (n = 283)	Controls (n = 3415)	p-value
Age	65 (58, 73)	54 (42, 66)	< 0.001	65 (58, 73)	58 (49, 68)	< 0.001
Women	203 (64%)	23,610 (55%)	0.001	180 (64%)	2053 (60%)	0.25
Body mass index (kg/m^a^)	27.5 (24.6, 30.4)	26.7 (24.1, 29.6)	0.015	27.5 (24.6, 30.5)	27.3 (24.4, 27.3)	0.72
Total cholesterol (mmol/L)	5.5 (4.8, 6.3)	5.4 (4.7, 6.2)	0.40	5.5 (4.8, 6.3)	5.6 (4.8, 6.4)	0.27
HDL cholesterol (mmol/L)	1.4 (1.1, 1.6)	1.3 (1.1, 1.6)	0.017	1.3 (1.1, 1.6)	1.3 (1.1, 1.6)	0.03
Diabetes	24 (8%)	1870 (4%)	0.018	23 (8%)	206 (6%)	0.36
Hypertension	192 (61%)	16,160 (37%)	< 0.001	172 (61%)	1570 (46%)	< 0.001
Smoking status			< 0.001			0.002
Never	107 (34%)	20,784 (48%)		101 (36%)	1510 (44%)	
Previous	131 (42%)	13,341 (31%)		118 (42%)	1088 (32%)	
Present	78 (25%)	9116 (21%)		64 (23%)	817 (24%)	
Creatinine (μmol/L)	84 (73, 93)	82 (73, 92)	0.20	84 (73, 93)	81(71, 92)	0.22
Time at risk (years)	11.1 (10.4, 11.6)	11.2 (10.8, 11.6)	< 0.001	11.1 (10.4, 11.6)	11.1 (10.7, 11.6)	0.029
Total observation time (years)	3146.6	465,701.7		2841.0	36,092.4	
Observed all-cause mortality^a^	85 27% (21%, 33%)	4705 11% (11%, 11%)	< 0.001	75 27% (21%, 33%)	492 14% (13%, 16%)	< 0.001
Causes of death			0.47			
Cancer	27 (32%)	1484 (31%)				
Circulatory disease	17 (20%)	1269 (27%)				
Respiratory disease	6 (7%)	319 (7%)				
Other	35 (41%)	1633 (35%)				
C-reactive protein (mg/L)	2.5 (1.1, 6.7)	1.2 (0.6, 2.7)	< 0.001	2.4 (1.1, 6.7)	1.5 (0.7, 3.2)	< 0.001
C-reactive protein concentration ≥ 3 mg/L	146 (46%)	9885 (23%)	< 0.001	128 (45%)	949 (28%)	< 0.001
Lactoferrin (μg/L)	NA	NA		500 (280, 949)	333 (222, 486)	< 0.001
Neopterin (nmol/L)	NA	NA		5.9 (4.3, 7.8)	5.1 (3.7, 6.7)	< 0.001
Age at RA diagnosis (years)	55 (45, 64)	NA		55 (45, 63)	NA	
Years with RA at HUNT3	9 (5, 16)	NA		9 (5, 16)	NA	
Seropositive^b^	227 (72%)	NA		201 (71%)	NA	

Data are given as number (%) or median (25th, 75th percentile). RA patients and controls were compared using the Chi-square test or Mann–Whitney U-test.

HDL, high-density lipoprotein; HUNT3, 3rd wave of the Trøndelag Health Study; NA, not applicable; RA, rheumatoid arthritis.

^a^95% confidence intervals in parenthesis, calculated using the binomial distribution.

^b^Seropositive: Positive for IgM rheumatoid factor and/or anti-citrullinated protein peptide antibodies.

### Cox regression analysis of mortality

Table 2 shows results from the Cox regression analyses, in which age differences between RA patients and controls were accounted for using age as time variable and sex was included as a stratification variable. In the main study, both RA and CRP ≥ 3 mg/L were significantly associated with all-cause mortality in the Step1 models and the Step2 models (additionally adjusted for body mass index, diabetes, hypertension, smoking and total cholesterol). HR became slightly lower following adjustment but were still clearly within the confidence intervals from the Step1 models. Thus, both variables were independently associated with mortality when tested separately. In the Step3 model including both RA and CRP, the HR for CRP hardly changed compared to the Step2b model and there was no significant interaction between RA and CRP (p = 0.64). However, the HR for RA fell from 1.34 in the Step2a model to 1.25 and RA was now barely significant (p = 0.048), consistent with the hypothesis that inflammation as captured by CRP may be a mediator of the increased mortality associated with RA.

**Table 2:** Cox regression models for all-cause mortality.

Model^a^	Variable	Hazard ratio (95% CI)	p-value
**Main analysis (n = 43,241)**			
Model 1a^b^	RA	1.42 (1.14, 1.77)	0.002
Model 1b^b^	CRP ≥ 3 mg/L	1.58 (1.49, 1.68)	< 0.001
Model 2a^c^	RA	1.34 (1.08, 1.67)	0.009
Model 2b^c^	CRP ≥ 3 mg/L	1.51 (1.42, 1.60)	< 0.001
Model 3^d^	RA	1.25 (1.00, 1.56)	0.048
	CRP ≥ 3 mg/L	1.50 (1.41, 1.60)	< 0.001
**Nested cohort analysis (n = 3698)**			
Model 4a^e^	RA	1.36 (1.06, 1.72)	0.014
Model 4b^e^	CRP ≥ 3 mg/L	1.64 (1.38, 1.94)	< 0.001
Model 4c^e^	Lactoferrin^g^	1.16 (1.03, 1.31)	0.018
Model 4d^e^	Neopterin^g^	1.21 (1.05, 1.40)	0.010
Model 5^f^	RA	1.16 (0.89, 1.50)	0.27
	CRP ≥ 3 mg/L	1.51 (1.26, 1.80)	< 0.001
	Lactoferrin^g^	1.07 (0.94, 1.21)	0.29
	Neopterin^g^	1.17 (1.01, 1.36)	0.031

CI, confidence interval; CRP, C-reactive protein; RA, rheumatoid arthritis.

^a^All models accounted for differences in age by using age as the time variable, and allowed for different baseline hazards in men and women by stratification on sex.

^b^Models 1a and 1b included either RA or CRP, respectively.

^c^Models 2a and 2b included either RA or CRP and additional adjustments for body mass index, diabetes, hypertension, smoking, and total cholesterol.

^d^Model 3 included RA and CRP and the adjustments mentioned above.

^e^Model 4a, 4b, 4c, and 4d, included either RA, CRP, Lactoferrin, or Neopterin, respectively.

^f^Model 5 included RA, CRP, Lactoferrin, and Neopterin, and the adjustments mentioned above.

^g^Transformed using natural logarithm.

### Mediation analysis of mortality

This hypothesis was further supported by the mediation analysis. The overall excess relative risk for all-cause mortality of having RA was 38% (7%, 68%, p = 0.015). The indirect effect of having CRP ≥ 3 mg/L mediated 10% (7%, 13%, p < 0.001, dashed arrows in Fig. 2b), whereas the direct effect of RA was 29% (− 7%, 68%, p = 0.12, solid arrow in Fig. 2b). The wide CI are probably related to the low number of RA cases. There was no significant interaction (− 2% (− 29%, 26%), p = 0.91), as in the Cox regression analysis.

### Sensitivity analyses

The results from the Step3 sensitivity analysis using CRP ≥ 5 mg/L as cut-off were essentially the same as with CRP ≥ 3 mg/L (RA; HR = 1.23 (0.99, 1.55), p = 0.062, CRP; HR = 1.58 (1.47, 1.70), p < 0.001). The change in significance for RA may be a false-negative result because the number of RA patients with CRP ≥ 5 mg/L (n = 89, i.e., 31%) was lower than the number with CRP ≥ 3 mg/L (n = 128, i.e., 45%). In the sensitivity analysis including both RA and CRP ≥ 3 mg/L without adjustment for classical CV risk factors, the HR were 1.29 (1.03, 1.62, p = 0.025) for RA and 1.57 (1.48, 1.67, p < 0.001) for CRP. Compared to the adjusted Step3 model, these results indicate that the influence of the classical CV risk factors on the associations of RA and CRP on mortality was small. There were no changes in the HR for RA nor CRP in the Step3 model including additional adjustments for previous cancer or chronic respiratory disease.

### Nested cohort study including CRP, lactoferrin and neopterin

In the nested cohort study, Cox regression showed that RA, CRP ≥ 3 mg/L, lactoferrin, and neopterin were all significantly associated with mortality when tested separately in adjusted models (Models 4a, 4b, 4c, and 4d, Table 2). When RA and all three biomarkers were included in the same model, CRP and neopterin remained significant, whereas RA and lactoferrin were not. The HR for RA changed from 1.36 to 1.16, consistent with inflammation as indicated by CRP and neopterin being mediators of increased mortality associated with RA.

Model fit including proportionality of hazards was good or acceptable for all Cox regression models. Within the main study and the nested cohort study, respectively, the Step3 and Step5 models had best fit.

## Discussion

In this large population-based study, RA and inflammation as captured by CRP and neopterin were significantly associated with all-cause mortality. Mediation analysis showed that the overall excess relative risk for all-cause mortality of having RA was 38%, and approximately a quarter of this was mediated by inflammation. Thus, other factors related to RA accounted for a substantial part of the excess mortality risk, even when adjusting for classical CV risk factors. The novelty of the study lies in this quantification of the impact on mortality mediated by inflammation.

Our results beg the question of what the main RA-associated mortality risk factors are in addition to inflammation. One possibility is a genetic component. However, we have recently shown that genetic risk factors for coronary artery disease have similar associations with future myocardial infarctions in RA patients and controls, and that variants predisposing to RA probably do not substantially increase the risk of myocardial infarctions^25^. These findings are important because CVD is one of the most frequent causes of death in RA, observed in 36.6% of patients in a large recent study^4^. To our knowledge, similar genetic investigations related to other important causes of death in RA like cancer have not been performed.

Another possibility is physiological or metabolic factors that were not adequately represented in our analysis. For example, a systematic review found strong evidence for reduced cardiac parasympathetic nervous activity in RA^26^, and traditional risk factors like lipids and systolic blood pressure are modulated by the inflammation of RA^11,27^. We have also shown that low cardiorespiratory fitness is an important mediator of RA-associated excess mortality^6^. The relative importance of such factors is unknown, and they are probably also interwoven and partly overlapping^12^.

Our study confirmed that including other inflammatory markers than CRP helped to represent inflammation in a broader sense. Addition of neopterin was useful because the marker was significantly associated with mortality. Neopterin is not associated with RA disease activity^28^, which is the case for CRP. Coronary and carotid atherosclerotic plaques in RA patients more often are unstable than in controls^29,30^, which may have been captured by the neopterin measurements. Thus, neopterin may also be considered a marker of the results of inflammation. Our study was not aimed at risk prediction, so we are not advocating measurement of neopterin in RA patients for clinical reasons.

The study has important clinical implications. As mentioned in the introduction, CVD is an important cause of death in RA patients. However, RA treatment has mostly been aimed at limiting joint damage, which is of utmost importance and depends on reducing inflammation^6,12^. The effects of RA medications on CV risk factors vary from beneficial for some like methotrexate to detrimental for others like glucocorticoids^12^, even if the use of biologic disease-modifying anti-rheumatic drugs has recently been suggested to explain a trend towards reduced excess CVD in RA^2^. Effects on CV risk factors could be given more explicit weight when choosing among RA medications. Furthermore, non-pharmacologic methods to reduce inflammation are important. Examples are adequate diets with less saturated fats^31^, reduction of overweight and strength exercises to normalize body composition and reduce production of proinflammatory cytokines by adipose tissue^32^, and aerobic exercise to improve fitness and increase the release of anti-inflammatory cytokines^32^. Such non-pharmacologic interventions may also improve other CV risk factors like blood pressure and lipids. Using definitions of RA remission with a strong emphasis on achieving minimal levels of general inflammation may also be warranted.

The study has both strengths and limitations. Strengths of this study are that the results are from a large population-based cohort, and the diagnosis ascertainment from hospital records was more accurate than in most registry-based studies. Several important adjustment variables were available. However, self-reported data may be inaccurate. The level of missingness was low. Follow-up time was long, which may be important because mortality differences between RA patients and controls may become larger as time passes^33^. As in all population-based studies, the number of RA patients was relatively low. This made it impossible to perform a separate analysis in seropositive patients only, or to analyze associations between inflammation and causes of death in RA. Furthermore, data were only registered once, and the biomarkers and other variables could have changed during follow-up. A single CRP measurement does not reflect the accumulated burden of inflammation, which probably has a stronger association with mortality. However, including further CRP measurements made after the start of the follow-up period would lead to methodological concerns. A study of non-participants in HUNT3 indicated that their frequency of musculoskeletal diseases was lower than that of participants, but that their mortality rate was higher^34^. We therefore cannot exclude that participant selection bias may have influenced our results.

Another possible limitation of our study is that CRP may not be a sufficiently good marker of inflammation, even if it is used clinically by rheumatologists to assess levels of inflammation and disease activity in RA patients. We cannot exclude that adding more or other inflammation markers than CRP in the main study would have reduced the unexplained part of the association with RA. Information about medication was not available. RA-specific medications would not have been useful in our analysis because of collinearity with presence or absence of RA, similar to other RA-specific variables like disease activity. Adjusting for use of other medications including drugs for CVD like statins could have been useful because prescription policies may differ between RA patients and controls.

In conclusion, the present study showed that approximately a quarter of the excess relative mortality risk of RA was mediated by inflammation. The findings underscore the importance of employing all available modalities to reduce inflammation, including carefully selected medications and lifestyle changes. Using definitions of RA remission with emphasis both on joint status and the level of general inflammation may help guide the most efficient treatment regimens. However, reducing inflammation alone cannot be expected to abolish excess mortality rates in RA.

## Data Availability

Data from the HUNT Study are available upon reasonable request from the HUNT Research Centre (https://www.ntnu.edu/hunt/data), following approval from the Regional Research Ethics Committee. However, restrictions apply to the availability of the data for the present paper, which were used under license for the current study and are not publicly available, in accordance with Norwegian law.
